# Design and synthesis of an erdafitinib-based selective FGFR2 degrader

**DOI:** 10.3762/bjoc.22.44

**Published:** 2026-04-15

**Authors:** Yumeng Jin, Shidong Wang, Sihan Pan, Shuqi Huang, Weichen Zhou, Xiaohao Huang, Lei Zheng, Lingfeng Chen

**Affiliations:** 1 School of Pharmacy, Hangzhou Medical College, Hangzhou 310012 Zhejiang, China

**Keywords:** CRBN, erdafitinib, FGFR2, selective degrader

## Abstract

This study aimed to develop a novel degrader capable of selectively degrading fibroblast growth factor receptor 2 (FGFR2) to overcome the issues of drug resistance and adverse reactions associated with traditional inhibitors in the treatment of FGFR2-driven tumors. Erdafitinib was employed as the targeting ligand, and its aliphatic amine site was conjugated with a CRBN E3 ligase ligand to design and synthesize a series of PROTAC molecules with different linkers. Screening was performed in KATO III cells with high FGFR2 expression, leading to the identification of LC-JD-6 as a potent degrader. Experimental results demonstrated that LC-JD-6 effectively induced FGFR2 protein degradation with a half-maximal degrading concentration (DC_50_) of 121.4 nM, and this effect exhibited time- and concentration-dependence. Assessed at the cellular level, LC-JD-6 has a half-maximal inhibitory concentration in the KATO III (IC_50_) of 96.0 nM and showed low inhibitory activity in normal cells. Selectivity analysis revealed that LC-JD-6 specifically degraded FGFR2 with minimal impact on other FGFR subtypes. Further studies confirmed that LC-JD-6 also efficiently reduced the expression of FGFR2 on the cell membrane surface. In conclusion, this study successfully developed LC-JD-6, a novel FGFR2-selective degrader, and for the first time confirmed its ability to degrade the membrane-bound form of FGFR2. This work provides an innovative targeted protein degradation strategy for the treatment of FGFR2-driven tumors and holds significant potential for clinical application.

## Introduction

Fibroblast growth factor receptors (FGFR) are a family of single-pass transmembrane receptor tyrosine kinases (RTKs) localized on the cell surface that bind to fibroblast growth factors [1–3]. Dimerization and autophosphorylation of FGFRs are induced by their binding to ligands, which trigger intracellular signaling cascades to activate downstream substrates and pathways [4]. This process involves PLC γ-mediated PKC activation and pFRS2-induced activation of the PI3K/AKT and MAPK/ERK pathways, thereby regulating cell proliferation, differentiation, migration, and survival [5–8]. FGF/FGFR dysregulation is associated with various diseases, including cancers, skeletal disorders, and metabolic syndromes [9–10]. Among family members, tumorigenesis driven by FGFR2 activation is attributed to genetic amplification, mutations, and gene rearrangements or fusions [11–13]. Genetic amplification of FGFR2 leads to an excessive number of gene copies, resulting in over-expression of the FGFR2 protein on the cell surface. This over-abundance can cause the receptor to be constitutively active, continuously sending growth signals to the cell even without proper ligand binding, thus promoting uncontrolled cell proliferation [11,14]. Mutations in FGFR2 may alter its structure, enabling it to bypass normal regulatory mechanisms and activate downstream oncogenic pathways independently of external stimuli, which also contributes to tumor development. Gene rearrangements or fusions involving FGFR2 can create novel chimeric proteins with enhanced or aberrant signaling capabilities, further driving cells towards a malignant phenotype [13,15]. Notably, FGFR2 amplification and fusions are frequently detected in patients with advanced gastric cancer and cholangiocarcinoma [16–18]. Accordingly, research into therapeutics targeting FGFR2 has emerged as a major focus.

**Figure 1:**
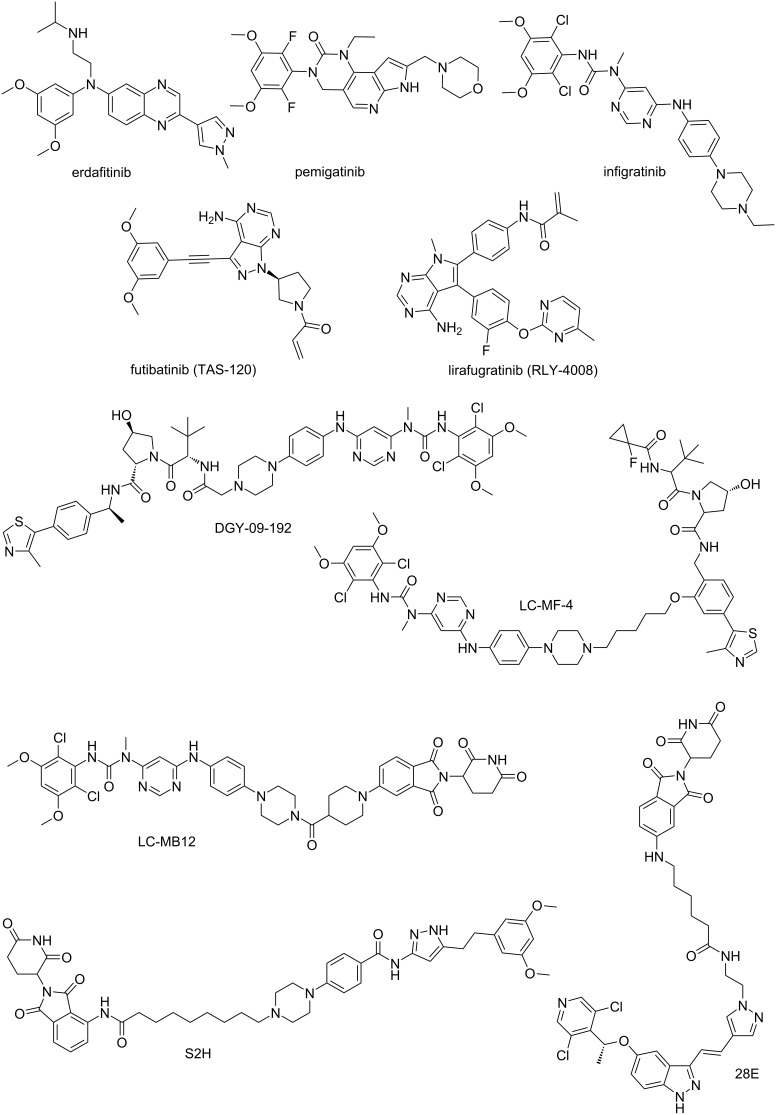
Chemical structures of severel FGFR inhibitors and degraders.

To treat FGFR2-driven tumors, current research primarily focuses on two distinct classes of inhibitors, namely FGFR pan-inhibitors and FGFR2-selective inhibitors. Among these in Figure 1, futibatinib, infigratinib, and erdafitinib, as pan-FGFR inhibitors, exhibit therapeutic efficacy against tumors driven by FGFR2. For advanced cholangiocarcinoma driven by FGFR2, infigratinib and futibatinib are targeted drugs specifically approved for this indication [19]. In contrast, erdafitinib is primarily effective in the treating of urothelial carcinoma with FGFR2/3 alterations [20]. However, due to the risk of adverse effects associated with pan-inhibitors, an increasing number of FGFR2-selective inhibitors have been reported in recent years [21–25]. Lirafugratinib (RLY-4008), an FGFR2-selective inhibitor, is the first highly selective FGFR2 inhibitor. Although substantial advancements have been made in developing FGFR2-targeted therapeutics, the sustained clinical efficacy of these inhibitors in oncology remains constrained by the emergence of acquired resistance mechanisms.

Proteolysis-targeting chimera (PROTAC) is a chemical molecule which induce the target protein to approach the ubiquitin protein through the ubiquitin proteasome system, then it can be ubiquitinated and degraded [26–28]. This drives to form degraders through the unique properties of their own degradation functions and overcome a series of critical issues in cancer treatment.

The numerous advantages of PROTAC technology in targeted therapy have prompted us to conduct further research into FGFR2 degraders. In this work, a series of degraders conjugating erdafitinib with a CRBN binder was synthesized and screened, leading to the identification of LC-JD-6, a potent and FGFR2-selective degrader. This study establishes a fundamental framework for the rational design of therapeutic modalities targeting FGFR2 degradation, offering novel insights into protein homeostasis-based cancer intervention.

## Results and Discussion

### FGFR2-targeted degrader design

**Figure 2:**
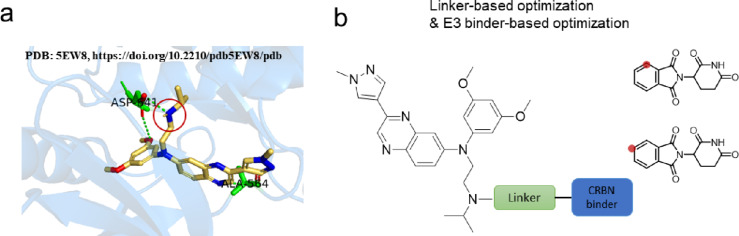
Rationale of the FGFR2 degrader design. a) Structure of the erdafitinib–FGFR complex. The figure was generated by PyMOL (PDB: 5EW8, https://doi.org/10.2210/pdb5EW8/pdb) [30]. b) The FGFR2 design strategy.

**Scheme 1:**
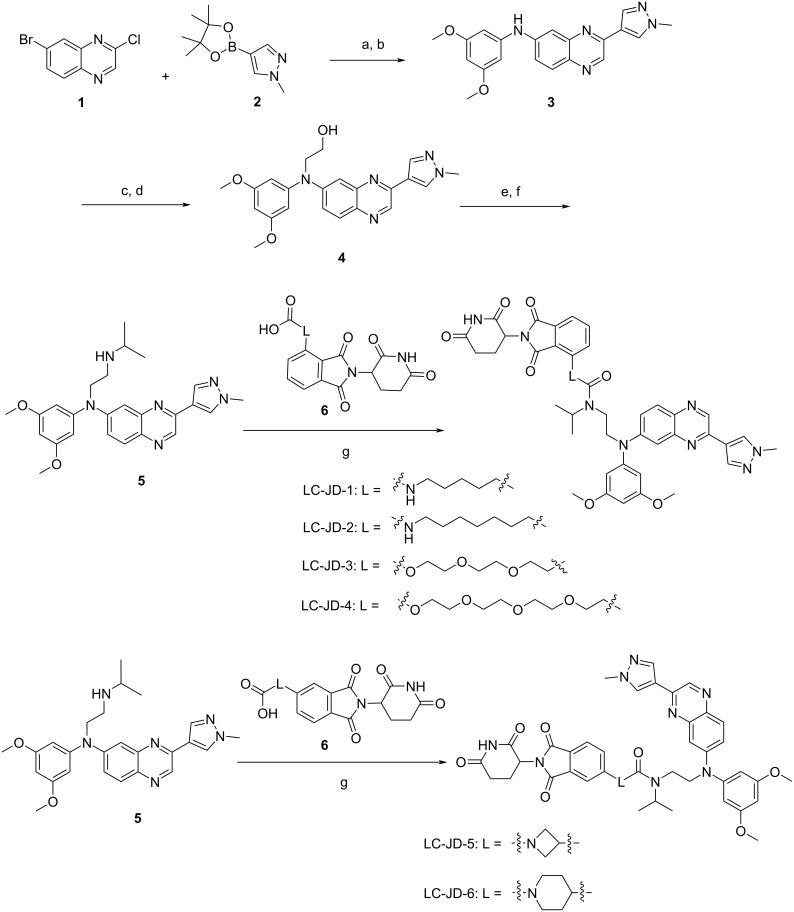
Synthesis of PROTACs towards FGFR2. Reagents and conditions: (a) K_2_CO_3_, Pd (dppf)Cl_2_, 1,4-dioxane/H_2_O 4:1, 100 °C, 5 h; (b) 3,5-dimethoxyaniline, Pd_2_(dba)_3_, BINAP, Cs_2_CO_3_, toluene, 100 °C, 12 h; (c) (2-bromoethoxy)-*tert*-butyldimethylsilane, NaH, DMF, rt, 12 h; (d) tetrabutylammonium fluoride, THF rt, 12 h; (e) methanesulfonic anhydride, TEA, DCM, rt, 3 h; (f) isopropylamine, DIPEA, MeCN, 100 °C, 12 h; (g) EDCI, HOBt, DIPEA, DMF, rt, 12 h.

PROTAC molecules are composed of three key elements: a specific ligand targeting the protein of interest (POI), a recruiter for an E3 ubiquitin ligase, and a linker that covalently connects these two functional moieties into a single molecular entity [29]. Herein, we purpose the clinically validated inhibitor erdafitinib as a POI binder that selectively targets FGFR2. Based on the analysis of the co-crystal structure of erdafitinib bound to the FGFR kinase domain (PDB: 5EW8, https://doi.org/10.2210/pdb5EW8/pdb) [30], we observed that the aliphatic amine group is positioned in the solvent-exposed region of the molecule. Given this observation, we selected the aliphatic amine group as a suitable conjugation site (Figure 2a). Previous studies have shown that different linkers exhibit distinct selectivity profiles [31]. Therefore, in this study, novel FGFR2 degraders were designed by conjugating the aliphatic amine in erdafitinib to two sites on CRBN (Figure 2b and Scheme 1), and the length and type of the linker were modified to achieve the selective degradation of FGFR2. Compound **5** was prepared according to the previously reported procedure [32–33], and then condensed with the reported compound 6 [31] under EDCI to afford the final novel FGFR2 degrader.

### Screening and identification of LC-JD-6 as a potent FGFR2 degrader

**Figure 3:**
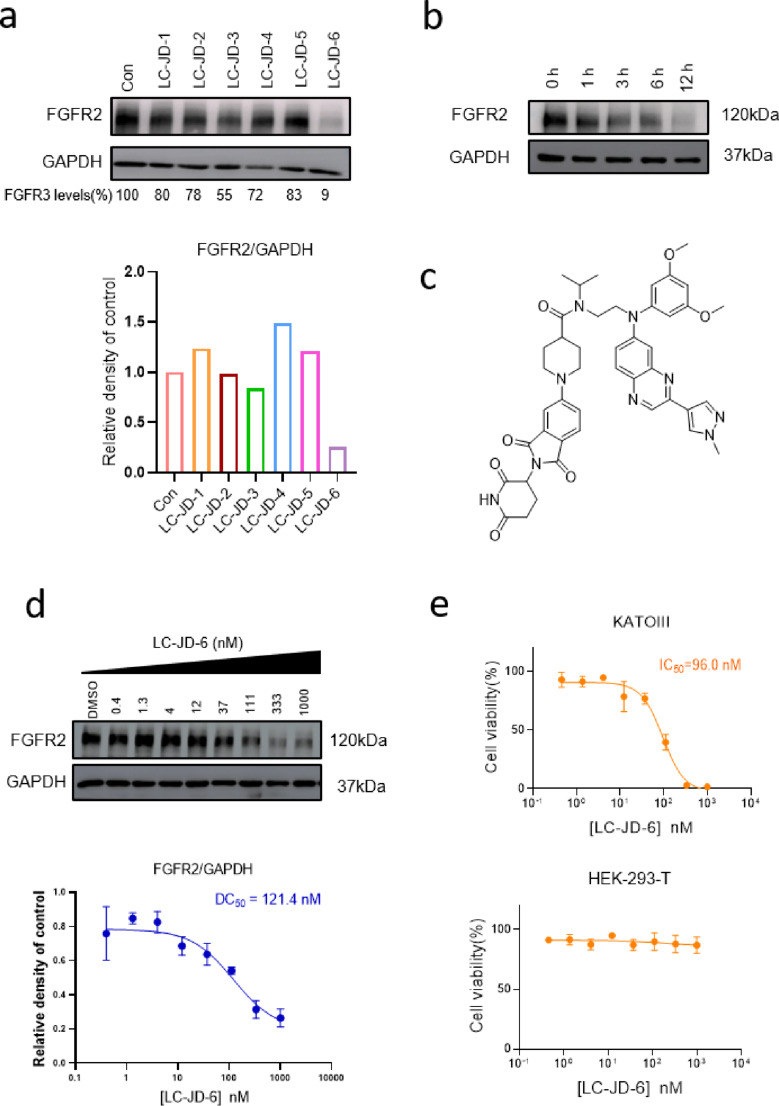
a) Representative western blots evaluating the total FGFR2 levels in KATO III cells following treatment using the indicated PROTAC. b) Time-course of FGFR2 degradation. c) Chemical structure of LC-JD-6. d,) Dose-course of FGFR2 degradation. e) Cell viability in KATO III and HEK293T cells.

Targeted protein degradation molecules in degrading FGFR2 were tested in KATO III cells (high basal FGFR2), identifying LC-JD-6 as the optimal candidate. Western blot analysis showed that LC-JD-6 reduced FGFR2 by 90% after 12 hours at 500 nM (Figure 3a). However, for structural modifications, neither long-chain alkyl linkers (LC-JD-1/2) nor PEG-based linkers (LC-JD-3/4) demonstrated significant degradation of FGFR2 protein levels in KATO III gastric cancer cells. Although LC-JD-5, which is the homoisomer of LC-JD-6, is still lacking compared to LC-JD-6. To further verify the potency, LC-JD-6 was prescribed at an extensive concentration range (0.4–10,00 nM) in KATO III cells after 6 hours, with the DC_50_ of 121.4 nM and a maximal degradation (D_max_) after 12 hours of treatment (Figure 3b and Figure 3d). As shown in Figure 3e, LC-JD-6 have potency in the KATO III proliferation assay with an IC_50_ of 96.0 nM and showed no significant inhibitory activity in HEK293T cells with low FGFR2 expression. Those results showed LC-JD-6 is indeed a potent degrader.

### LC-JD-6 as a selective degrader for FGFR2

**Figure 4:**
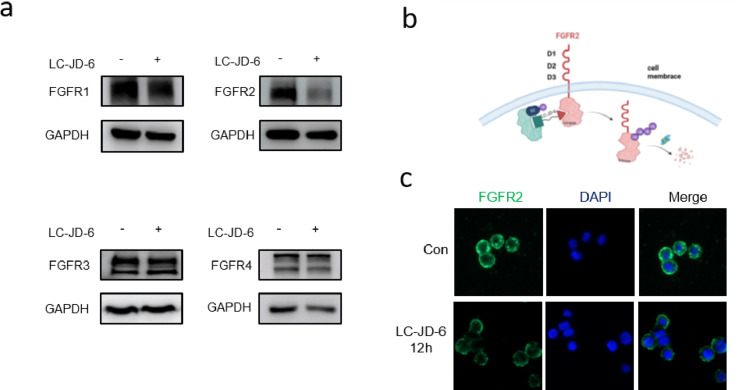
a) FGFR1, FGFR2, FGFR3, and FGFR4 levels in cells after treatment. b) The mechanism of PROTAC. It was created in BioRender (Chen, Lingfeng https://BioRender.com/7td2yot). This content is not subject to CC BY 4.0. c) Cellular localization of FGFR2 after treatment with LC-JD-6.

Pharmacologically, the capacity of PROTACs to discriminate between intended and unintended targets represents a pivotal attribute that mitigates adverse off-target effects. Therefore, LC-JD-6 underwent in vitro profiling against diverse FGFR subtypes to evaluate its potential as a selective degrader. The selective induction of FGFR2 degradation by LC-JD-6 was demonstrated in cell lines endogenously expressing four FGFR subtypes (NCI-H1581, KATO III, RT112, and Hep3B). As shown in Figure 4a, treatment with 100 nM LC-JD-6 for 12 hours resulted in a reduction of over 80% in FGFR2 protein levels, with negligible effects on the other subtypes. These findings establish LC-JD-6 as a highly selective FGFR2 degrader.

### LC-JD-6 reduced the expression of membrane-bound FGFR2

Since most PROTACs reported to date act on intracellular targets, we examined whether LC-JD-6 could directly degrade FGFR2 from the plasma membrane. KATO III gastric cancer cells were incubated with 500 nM LC-JD-6 for 12 hours, followed by flow cytometric quantification of cell surface FGFR2 expression. As shown in Figure 4b and Figure 4c, LC-JD-6 treatment led to a marked decrease in cell surface FGFR2 expression, supporting its role as a membrane-specific degrader.

## Conclusion

Over the past decade, with the identification of FGFR2 as a key oncogenic driver in multiple malignancies [34], significant progress has been made in elucidating the molecular mechanisms underlying the FGFR2 signaling pathway [35–36]. These advances have driven the development of several selective FGFR2 inhibitors, including erdafitinib, infiglatinib, and FGF401 [37–39]. However, issues such as acquired drug resistance and on-target toxicity emerging in clinical applications have limited the long-term efficacy of such inhibitors, and some of these agents remain in clinical development or have been discontinued [21–22]. Against this backdrop, the PROTAC technology has offered a novel strategy to overcome these challenges. Previously reported degraders, such as LC-MB12 [28], have been shown to effectively degrade FGFR2 via the proteasomal pathway, while the VHL E3 ligase-based degrader DGY-09-192 is capable of degrading both FGFR1 and FGFR2 simultaneously [40]. These findings suggest that different E3 ligase ligands may influence the selectivity and efficiency of degradation [41]. Nevertheless, due to limitations in experimental conditions, the specific regulatory mechanisms by which E3 ligase components modulate receptor selectivity remain to be further elucidated.

In general, the PROTAC technology has opened up a new avenue for addressing the drug resistance of traditional inhibitors by harnessing the intracellular endogenous protein degradation machinery. However, this technology still faces challenges in degrading membrane proteins such as FGFR2, partly due to the cell-type-specific expression differences of E3 ligases. Through systematic medicinal chemistry design and pharmacodynamic evaluation, this study successfully developed LC-JD-6, a CRBN E3 ligase-based PROTAC molecule that potently and selectively degrades FGFR2. Experiments demonstrated that LC-JD-6 exhibited a DC_50_ of 121.4 nM in KATO III cells, degraded more than 90% of FGFR2 protein within 12 hours, and displayed favorable subtype selectivity and the ability to degrade membrane-bound FGFR2. This molecule successfully repurposed erdafitinib, a broad-spectrum FGFR inhibitor, into a highly selective degrader. It not only provides a new chemical tool for investigating FGFR2 function but also lays a crucial foundation for the development of novel therapeutic strategies against FGFR2-driven tumors.

## Supporting Information

File 1 Experimental details and spectral data for all compounds.

## Data Availability

Data generated and analyzed during this study is available from the corresponding author upon reasonable request.
