# Supplementary material for: Design and synthesis of an erdafitinib-based selective FGFR2 degrader
Source: Beilstein J Org Chem. 2026 Apr 15;22:583–91. doi: 10.3762/bjoc.22.44 (PMC13159326; doi:10.3762/bjoc.22.44)
Supplement: File 1 — Experimental details and spectral data for all compounds. [file Beilstein_J_Org_Chem-22-583-s001.pdf]

BEILSTEIN JOURNAL OF ORGANIC CHEMISTRY

Supporting Information

for

Design and synthesis of an erdafitinib-based selective FGFR2 degrader

Yumeng Jin, Shidong Wang, Sihan Pan, Shuqi Huang, Weichen Zhou, Xiaohao Huang, Lei Zheng and Lingfeng Chen

Beilstein J. Org. Chem. **2026,** *22,* 583–591. doi:10.3762/bjoc.22.44

Experimental details and spectral data for all compounds

License and Terms: This is a supporting information file under the terms of the Creative Commons Attribution License (https://creativecommons.org/licenses/by/4.0). Please note that the reuse, redistribution and reproduction in particular requires that the author(s) and source are credited and that individual graphics may be subject to special legal provisions.
The license is subject to the *Beilstein Journal of Organic Chemistry* terms and conditions: (https://www.beilstein-journals.org/bjoc/terms)

EXPERIMENTAL SECTION

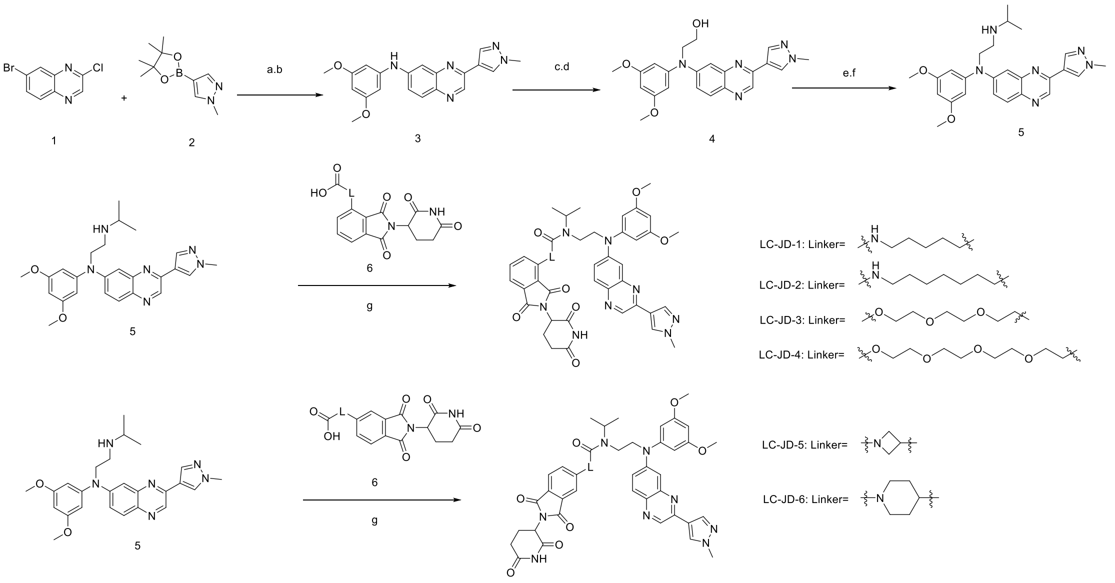

Figure S1: Reagents and conditions: (a) K_2CO_3, Pd (dppf)Cl_2, 1,4-dioxane: H_2O = 4:1, 100 °C,5 h; (b) 3,5-dimethoxyaniline, Pd_2 (dba)$_3$, BINAP, Cs_2CO_3, toluene, 100 °C, 12h; (c) (2-bromoethoxy)-*tert*-butyl-dimethylsilane, NaH, DMF, rt, 12 h; (d) tetrabutylammonium fluoride, THF rt, 12 h; (e) methanesulfonic anhydride, TEA, DCM, rt, 3 h; (f) isopropylamine, DIPEA, MeCN, 100 °C, 12 h.(g) EDCI, HOB, DIPEA, DMF, rt, 12 h.

1. Chemistry

All target compounds were purified via column chromatography with an elution system of DCM and methanol in different ratios, and their structures were confirmed via 1H nuclear magnetic resonance (NMR), ^{13}C NMR, and liquid chromatograph−mass spectrometry (LC-MS). High-resolution MS data were recorded using a Waters Qda accurate mass LC-Q/MS system KBF7798; Waters). ^{1}H and ^{13}C NMR spectral data were recorded on a 400 MHz spectrometer (Bruker Magnet System 400' 54 Ascend). Unless otherwise stated, all solvents or reagents were obtained from commercially available materials and used directly without further purification. Column chromatography was performed using a 200−300 mesh silica gel. The purities of all the synthesized compounds were >95% determined using the analytical HPLC on a Shimadzu LC-20AD system, Diamonsil C^{18} column.

1.1 *N-(2-((3,5-dimethoxyphenyl)(3-(1-methyl-1H-pyrazol-4-yl)quinoxalin-6-*

yl)amino)ethyl)-6-((2-(2,6-dioxopiperidin-3-yl)-1,3-dioxoisoindolin-4-yl)amino)-N-isopropylhexanamide (LC-JD-1). 1-(2-(2,6-dioxopiperidin-3-yl)-1,3-dioxoisoindolin-5-yl)piperidine-4-carboxylic acid (**6**, 50.1 mg, 0.13 mmol)，EDCI (53 mg, 0.14 mmol), HOBC (17.42mg,0.129 mmol), DIPEA (113 μL, 0.65 mmol) were dissolved in 3 mL DMF and stirred for 1 h. Then N^1-(3,5-dimethoxyphenyl)-N^2-isopropyl-N^1-(3-(1-methyl-1H-pyrazol-4-yl)quinoxalin-6-yl)ethane-1,2-diamine (**5**) (50mg, 0.13 mmol) were dissolved in and the reaction was stirred for 12h. After the reaction, diluted with H_2O, after extraction, the combined organic layer was concentrated and purified by chromatography on silica gel to yield the target compound: yellow powder (43%). ^{1}H NMR (400 MHz, DMSO-d_6) δ 11.10 (s, 1H), 8.97 (s, 1H), 8.54 (s, 1H), 8.20 (s, 1H), 7.80 (s, 1H), 7.56 (s, 1H), 7.43 (s, 1H), 7.31 (s, 1H), 7.04 (d, J = 33.6 Hz, 2H), 6.56 – 6.34 (m, 4H), 5.13 – 4.97 (m, 1H), 4.09 (s, 1H), 4.04 (d, J = 6.5 Hz, 3H), 3.60 – 3.22 (m, 6H), 2.87 (s, 1H), 2.38 (s, 1H), 1.98 (s, 3H), 1.60 (s, 3H), 1.30 – 1.22 (m, 1H), 1.17 (s, 5H), 1.06 (s, 7H), 0.85 (h, J = 9.9 Hz, 1H). ^{13}C NMR (101 MHz, DMSO) δ 173.30, 172.72, 170.59, 169.45, 167.81, 161.92, 149.24, 148.48, 147.64, 146.94, 143.98, 140.50, 138.34, 136.78, 136.24, 132.70, 131.30, 129.73, 120.83, 117.70, 110.87, 109.51, 104.20, 103.31, 97.17, 55.82, 50.69, 49.04, 48.10, 42.28, 40.66, 40.45, 40.24, 40.03, 39.83, 39.62, 39.41, 33.16, 31.48, 29.12, 26.63, 25.20, 22.66, 21.41, 20.73. LC-MS (ESI$^+$): calcd for $C_{44}H_{49}N_9O_7$ 815.9, 816.39 $[M + H]^+$.

LC-JD-1 ^{1}H NMR

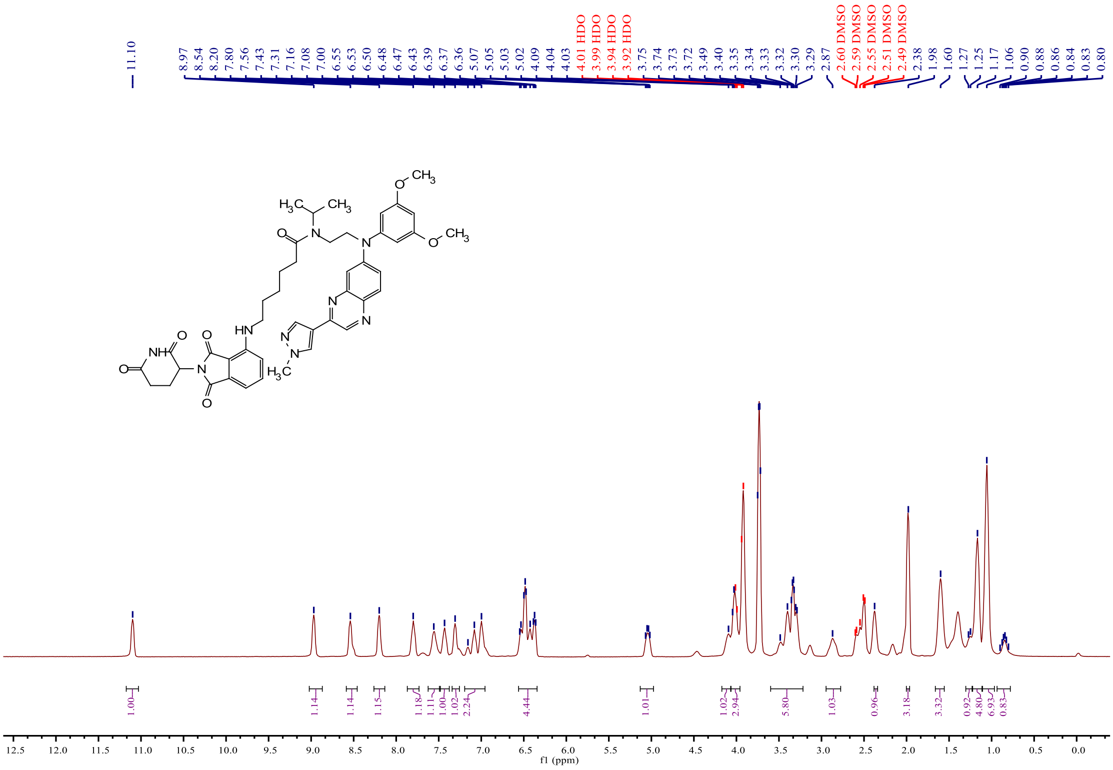

LC-JD-1 ^{13}C NMR

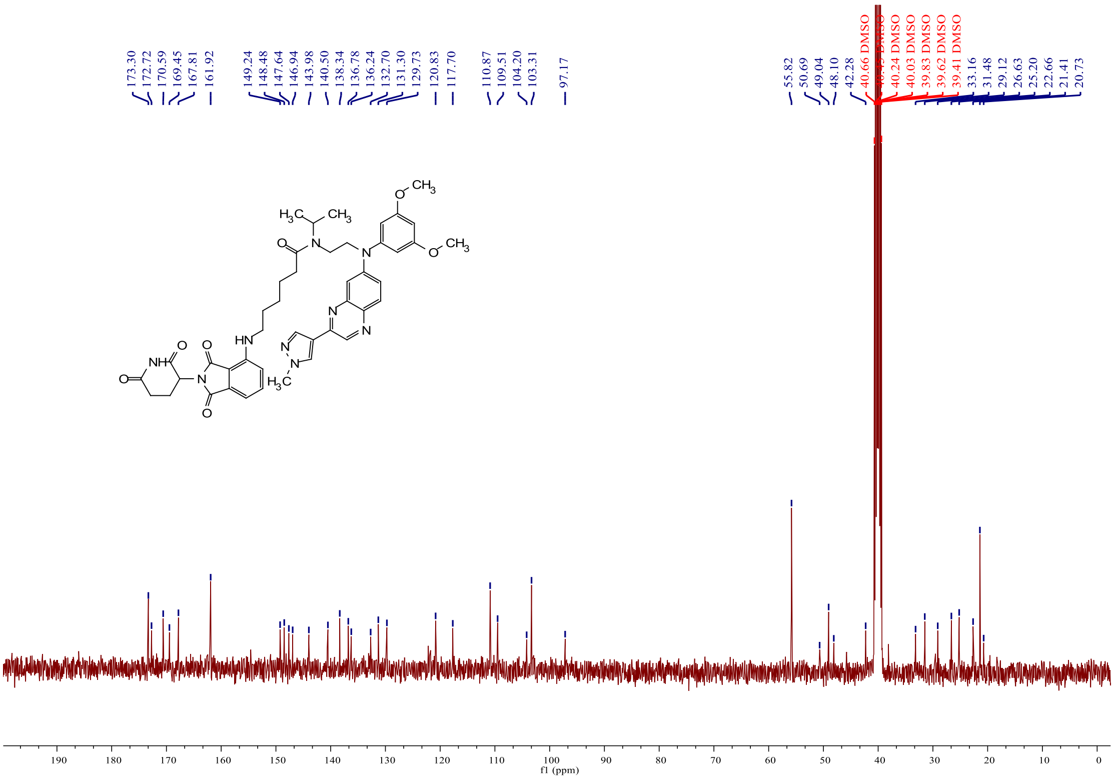

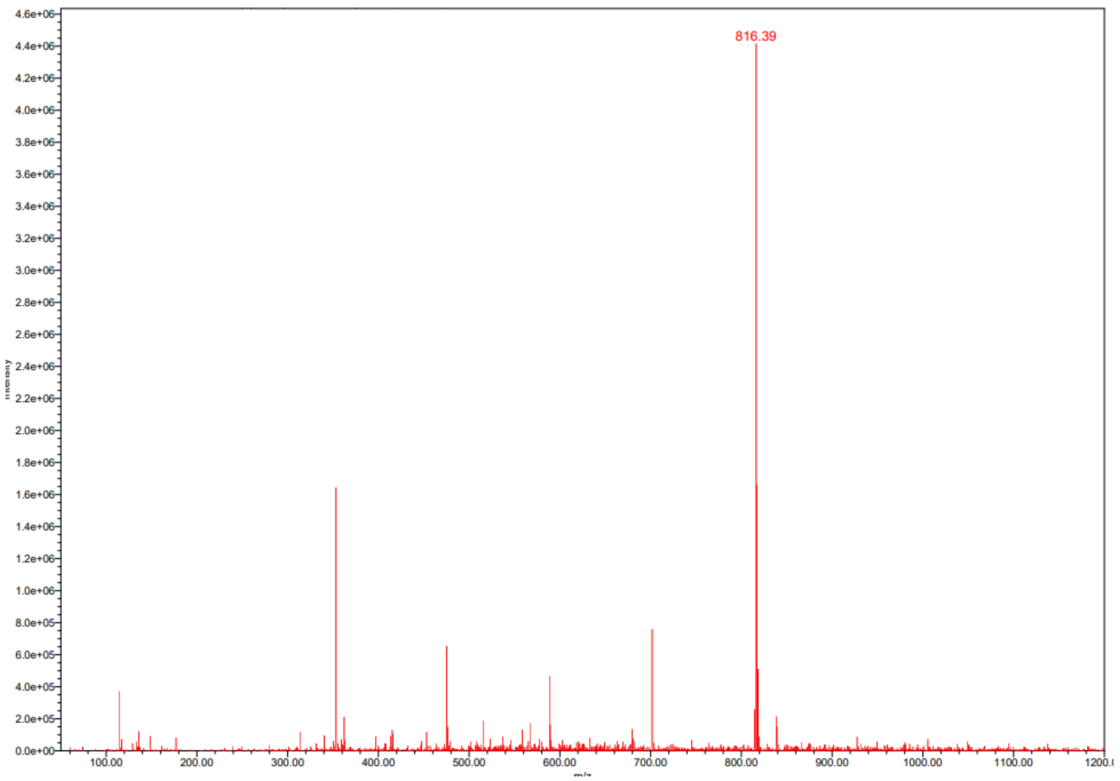

1.2 *N-(2-((3,5-dimethoxyphenyl)(3-(1-methyl-1H-pyrazol-4-yl)quinoxalin-6-yl)amino)ethyl)-8-((2-(2,6-dioxopiperidin-3-yl)-1,3-dioxoisoindolin-4-yl)amino)-N-isopropyloctanamide* (LC-JD-2). LC-JD-2 was synthesized in a similar manner as LC-JD-1, Yellow powder (53%). ^{1}H NMR (400 MHz, DMSO-d_6) δ 11.12 (s, 1H), 8.98 (d, J = 9.4 Hz, 1H), 8.55 (d, J = 9.8 Hz, 1H), 8.21 (d, J = 9.3 Hz, 1H), 7.82 (d, J = 9.3 Hz, 1H), 7.57 (s, 1H), 7.45 (d, J = 9.5 Hz, 1H), 7.32 (s, 1H), 7.04 (d, J = 22.3 Hz, 2H), 6.60 – 6.35 (m, 4H), 5.06 (s, 1H), 4.02 – 3.83 (m, 6H), 3.75 (d, J = 9.2 Hz, 6H), 3.52 – 3.17 (m, 6H), 2.89 (s, 1H), 2.36 (s, 2H), 2.22 – 1.84 (m, 3H), 1.57 (s, 4H), 1.34 (s, 5H), 1.19 (s, 4H). ^{13}C NMR (101 MHz, DMSO) δ 173.31, 172.83, 170.60, 169.46, 167.81, 161.92, 149.24, 148.49, 147.63, 146.93, 143.98, 140.50, 138.34, 136.77, 136.23, 132.69, 131.29, 129.72, 122.18, 117.68, 110.87, 109.50, 104.23, 103.30, 97.16, 55.82, 50.72, 49.05, 48.12, 42.34, 40.65, 40.44, 40.23, 40.02, 39.81, 39.60, 39.39, 38.13, 33.25, 31.49, 29.29, 29.16, 26.76, 25.45, 22.66, 21.41, 20.72. LC-MS (ESI^{+}): calcd for $C_{46}H_{53}N_9O_7$ 843.9, 844.44 $[M + H]^{+}$.

LC-JD-2 ^{1}H NMR

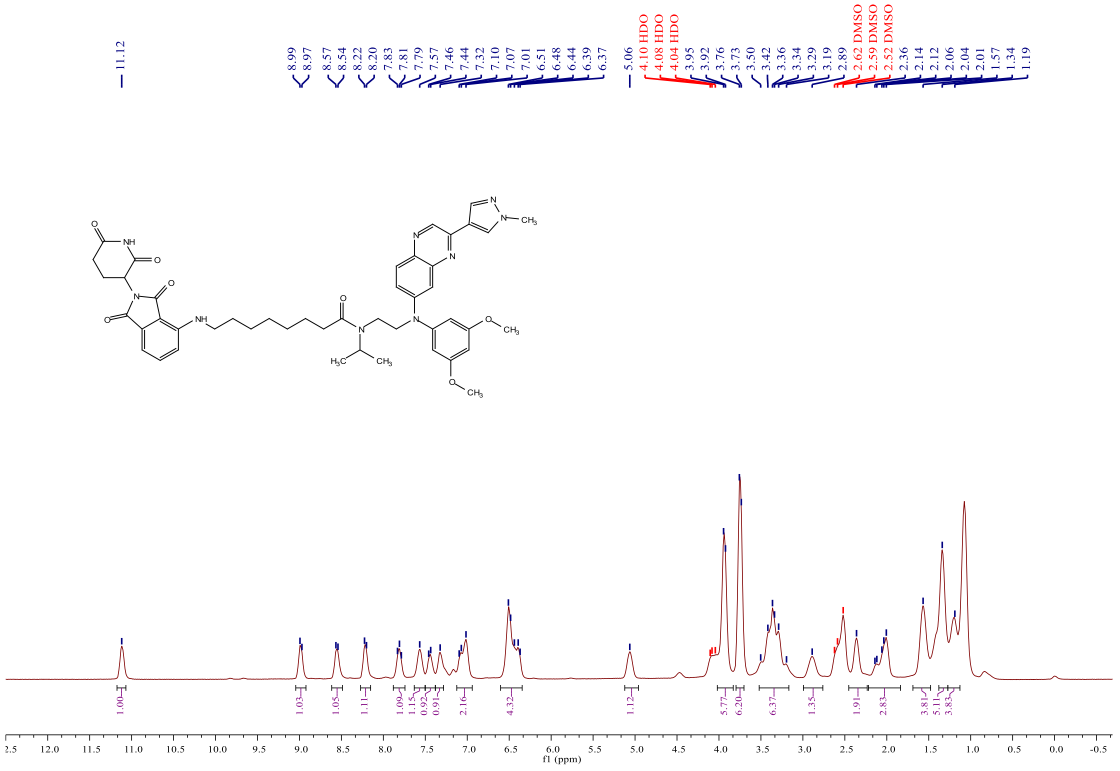

LC-JD-2 ^{13}C NMR

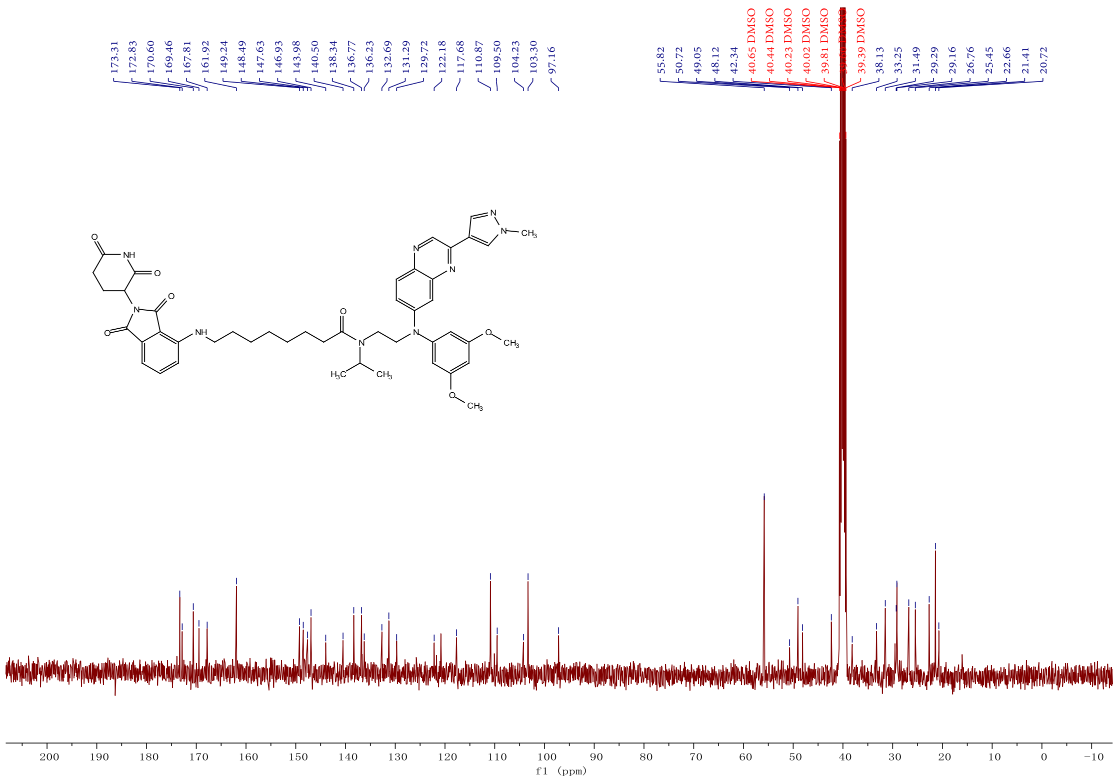

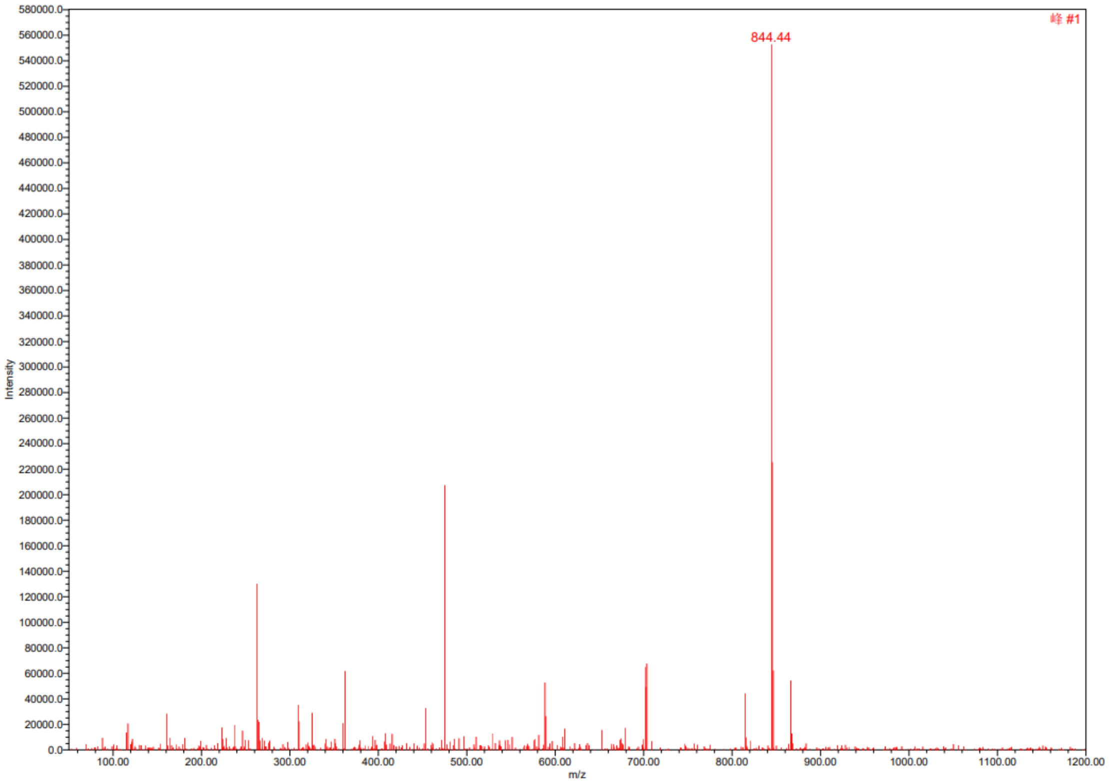

1.3 *N-(2-((3,5-dimethoxyphenyl)(3-(1-methyl-1H-pyrazol-4-yl)quinoxalin-6-yl)amino)ethyl)-3-(2-(2-((2-(2,6-dioxopiperidin-3-yl)-1,3-dioxoisoindolin-5-yl)oxy)ethoxy)ethoxy)-N-isopropylpropanamide* (LC-JD-3). LC-JD-3 was synthesized in a similar manner as LC-JD-1, Yellow powder (56%). ^{1}H NMR (400 MHz, DMSO-d_6) δ 11.17 (s, 1H), 9.05 (s, 1H), 8.62 (d, *J* = 8.2 Hz, 1H), 8.28 (d, *J* = 2.6 Hz, 1H), 7.89 (dd, *J* = 9.2, 3.3 Hz, 1H), 7.63 (dd, *J* = 8.5, 7.0 Hz, 1H), 7.50 (dd, *J* = 9.2, 2.7 Hz, 1H), 7.41 – 7.32 (m, 1H), 7.27 – 7.13 (m, 1H), 7.09 (dd, *J* = 7.1, 4.0 Hz, 1H), 6.54 (dd, *J* = 17.9, 2.2 Hz, 2H), 6.50 – 6.43 (m, 1H), 5.12 (dd, *J* = 12.9, 5.4 Hz, 1H), 4.20 (p, *J* = 6.5 Hz, 1H), 4.01 (s, 4H), 3.81 (d, *J* = 2.7 Hz, 6H), 3.76 (t, *J* = 6.7 Hz, 2H), 3.72 – 3.52 (m, 8H), 3.55 – 3.44 (m, 2H), 2.96 (ddd, *J* = 23.3, 11.8, 5.1 Hz, 1H), 2.71 (t, *J* = 6.9 Hz, 2H), 2.63 (d, *J* = 8.6 Hz, 1H), 1.45 – 1.28 (m, 1H), 1.31 (s, 1H), 1.15 (t, *J* = 6.7 Hz, 6H), 0.08 (s, 1H). ^{13}C NMR (101 MHz, DMSO) δ 173.31, 171.01, 170.58, 169.42, 161.92, 149.21, 148.45, 147.63, 146.87, 143.97, 140.50, 138.34, 136.68, 136.23, 132.57, 131.28, 129.73, 122.13, 120.83, 117.89, 111.15, 110.86, 109.73, 103.30, 97.18, 70.34, 70.30, 70.25, 69.37, 67.43, 55.82, 50.63, 49.05, 48.25, 42.18, 40.64, 40.43, 40.22, 40.01, 39.80, 39.59, 39.39, 38.10, 33.73, 31.49, 22.65, 21.32. LC-MS (ESI$^+$): calcd for $C_{45}H_{50}N_8O_{10}$ 871.39, 872.39 $[M + H]^+$.

LC-JD-3 ^{1}H NMR

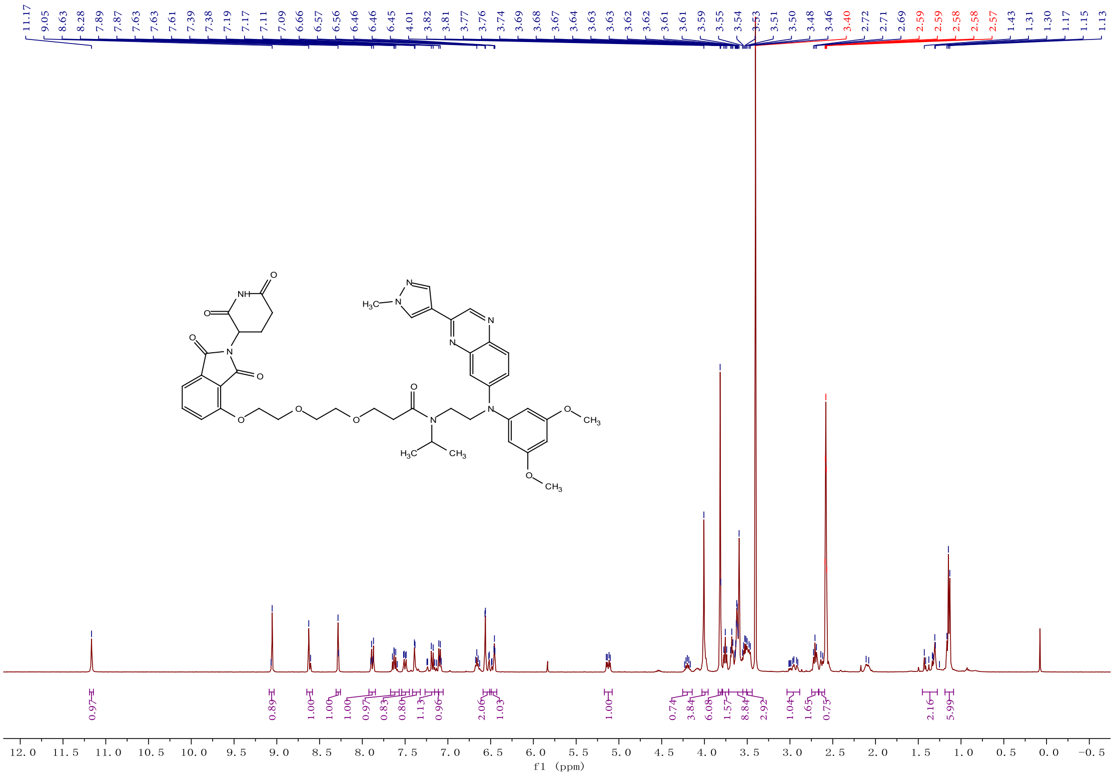

LC-JD-3 ^{13}C NMR

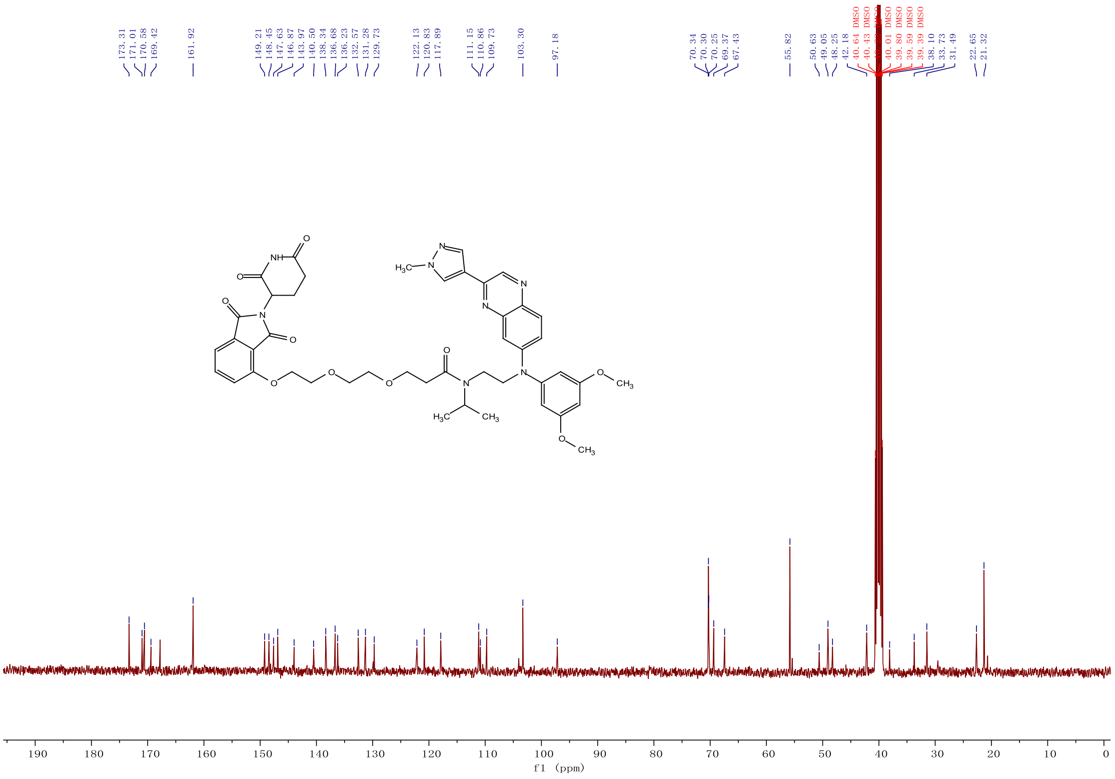

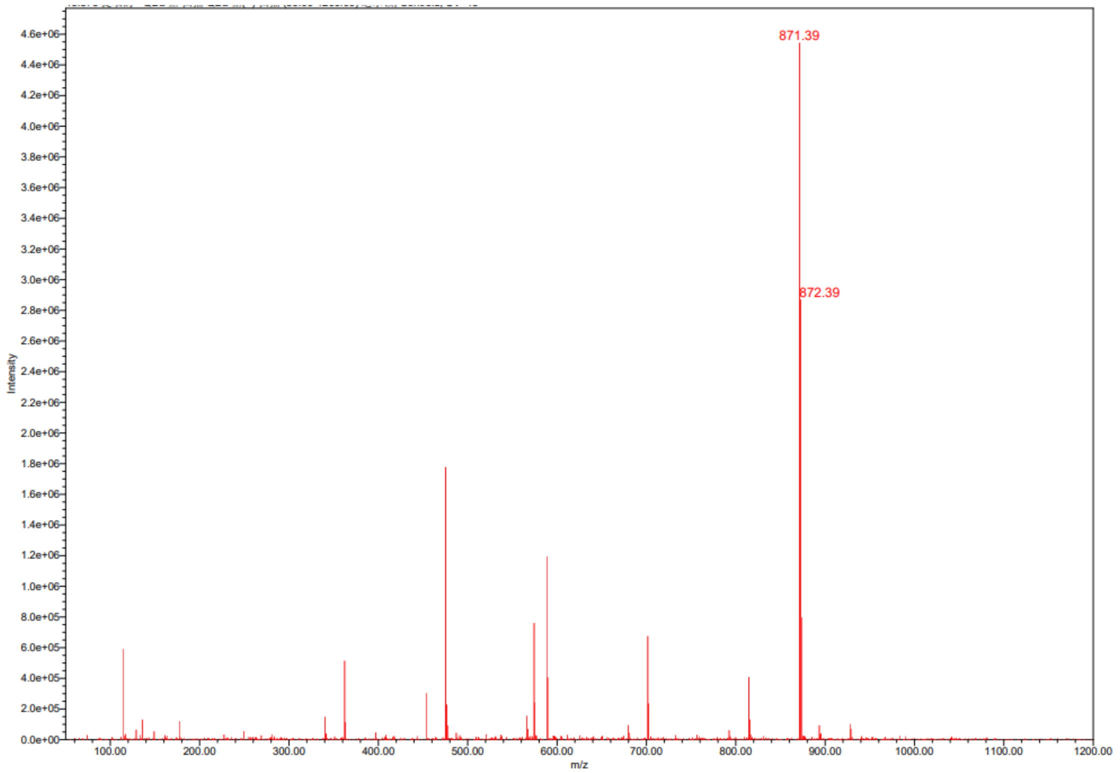

1.4 *N-(2-((3,5-dimethoxyphenyl)(3-(1-methyl-1H-pyrazol-4-yl)quinoxalin-6-yl)amino)ethyl)-4-(2-(2-(2-((2-(2,6-dioxopiperidin-3-yl)-1,3-dioxoisoindolin-5-yl)oxy)ethoxy)ethoxy)ethoxy)-N-isopropylbutanamide* (LC-JD-4). LC-JD-4 was synthesized in a similar manner as LC-JD-1, Yellow powder (60%). ^{1}H NMR (400 MHz, DMSO-d_6) δ 11.17 (s, 1H), 9.11 – 8.99 (m, 2H), 8.63 (s, 2H), 8.29 (s, 2H), 7.95 – 7.83 (m, 3H), 6.57 (d, J = 2.2 Hz, 1H), 6.55 (d, J = 2.2 Hz, 1H), 6.51 – 6.48 (m, 1H), 6.46 (dd, J = 5.4, 2.5 Hz, 1H), 4.01 (s, 6H), 3.83 (s, 3H), 3.82 (s, 6H), 3.61 (dd, J = 10.4, 5.2 Hz, 6H), 3.57 – 3.52 (m, 8H), 3.48 (dd, J = 10.0, 6.7 Hz, 4H), 1.21 (d, J = 6.7 Hz, 4H), 1.16 (d, J = 6.9 Hz, 4H). ^{13}C NMR (101 MHz, DMSO) δ 173.32, 170.59, 169.44, 167.80, 163.73, 161.93, 161.69, 149.07, 147.64, 146.91, 143.96, 138.36, 136.73, 132.60, 131.30, 120.82, 117.96, 103.27, 86.40, 70.29, 69.39, 55.82, 49.06, 40.64, 40.44, 40.23, 40.02, 39.81, 39.60, 39.39, 31.66, 31.49, 30.33, 29.53, 29.21, 22.65, 22.07, 21.32. LC-MS (ESI$^+$): calcd for $C_{47}H_{56}N_8O_{11}$ 906.47, 907.65.0 [M + H]$^+$.

LC-JD-4 ^{1}H NMR

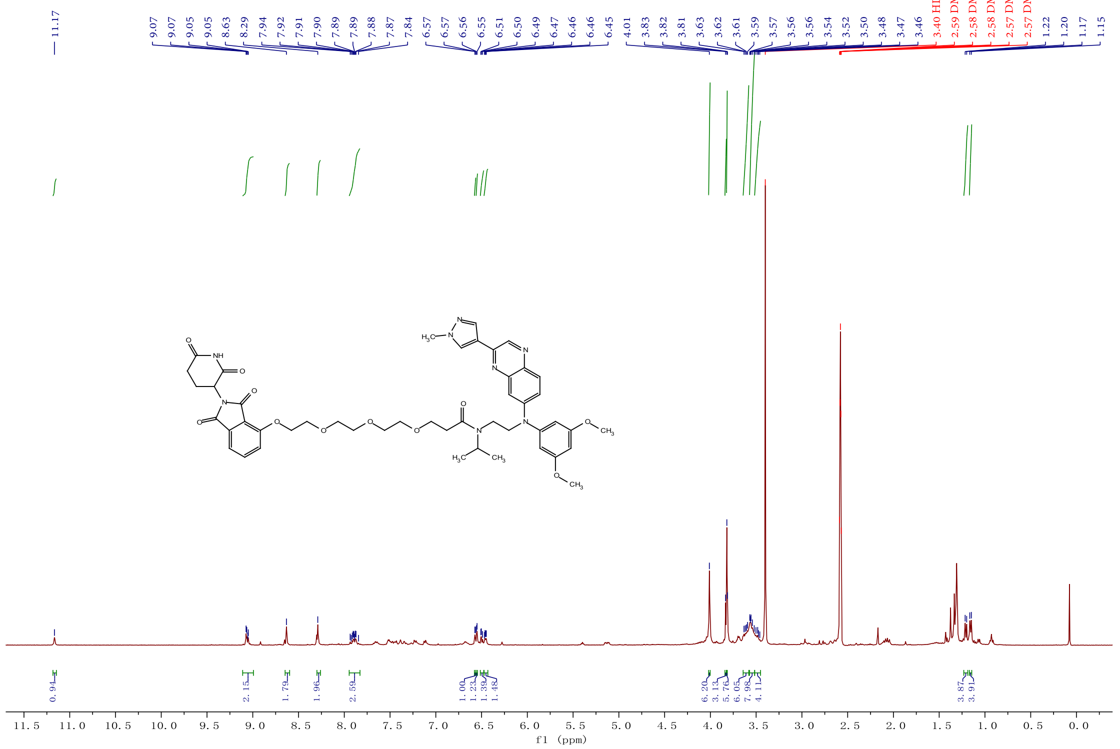

LC-JD-4 ^{13}C NMR

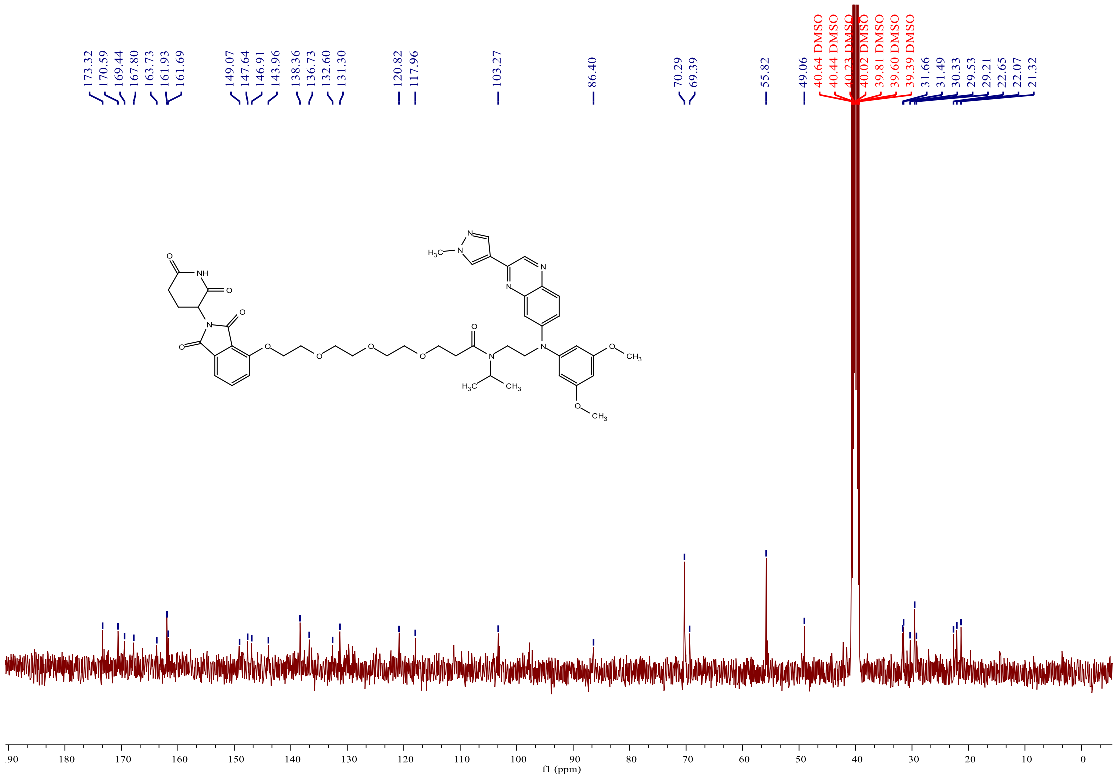

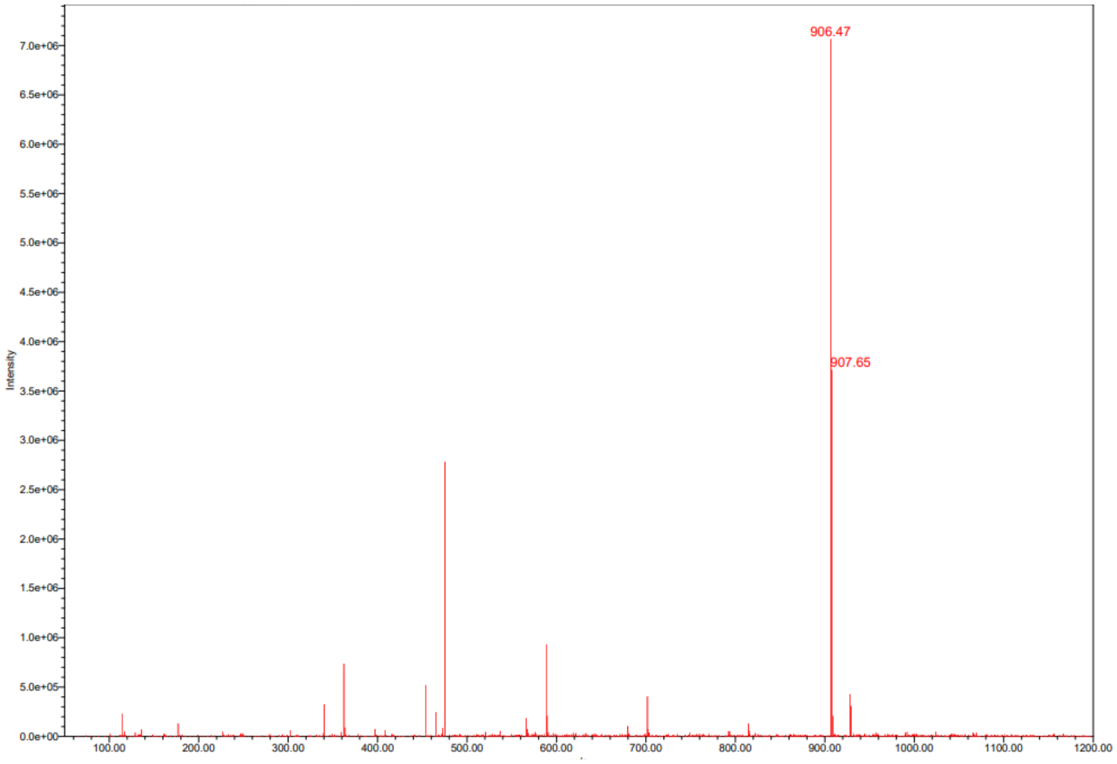

1.5 *N-(2-((3,5-dimethoxyphenyl)(3-(1-methyl-1H-pyrazol-4-yl)quinoxalin-6-yl)amino)ethyl)-1-(2-(2,6-dioxopiperidin-3-yl)-1,3-dioxoisoindolin-5-yl)-N-isopropylazetidine-3-carboxamide*（LC-JD-5）. LC-JD-5 was synthesized in a similar manner as LC-JD-1, yellow powder (62 mg,54%). ^{1}H NMR (400 MHz, DMSO-d_6) δ 11.09 (s, 1H), 8.96 (s, 1H), 8.53 (s, 1H), 8.19 (s, 1H), 7.81 (s, 1H), 7.65 (s, 1H), 7.43 (s, 1H), 7.34 (s, 1H), 6.83 (s, 1H), 6.69 (s, 1H), 6.49 (s, 2H), 6.38 (s, 1H), 5.15 – 5.00 (m, 1H), 4.20 (d, J = 39.7 Hz, 4H), 4.00 (s, 4H), 3.92 (s, 3H), 3.86 (d, J = 12.2 Hz, 1H), 3.73 (s, 6H), 3.33 (s, 3H), 2.89 (t, J = 19.5 Hz, 1H), 1.99 (s, 2H), 1.18 (s, 2H). ^{13}C NMR (101 MHz, DMSO) δ 173.31, 171.44, 170.61, 167.94, 167.67, 161.90, 155.44, 149.18, 148.55, 147.63, 143.95, 140.52, 138.31, 136.23, 134.27, 131.25, 129.70, 125.27, 122.22, 117.71, 114.81, 110.91, 103.26, 97.13, 55.81, 54.11, 49.23, 48.26, 40.64, 40.43, 40.23, 40.02, 39.81, 39.60, 39.39, 32.18, 31.50, 29.53, 21.38. LC-MS (ESI$^+$): calcd for $C_{42}H_{43}N_9O_7$ 785.6, found,786.29 [M + H]$^+$.

LC-JD-5 ^{1}H NMR

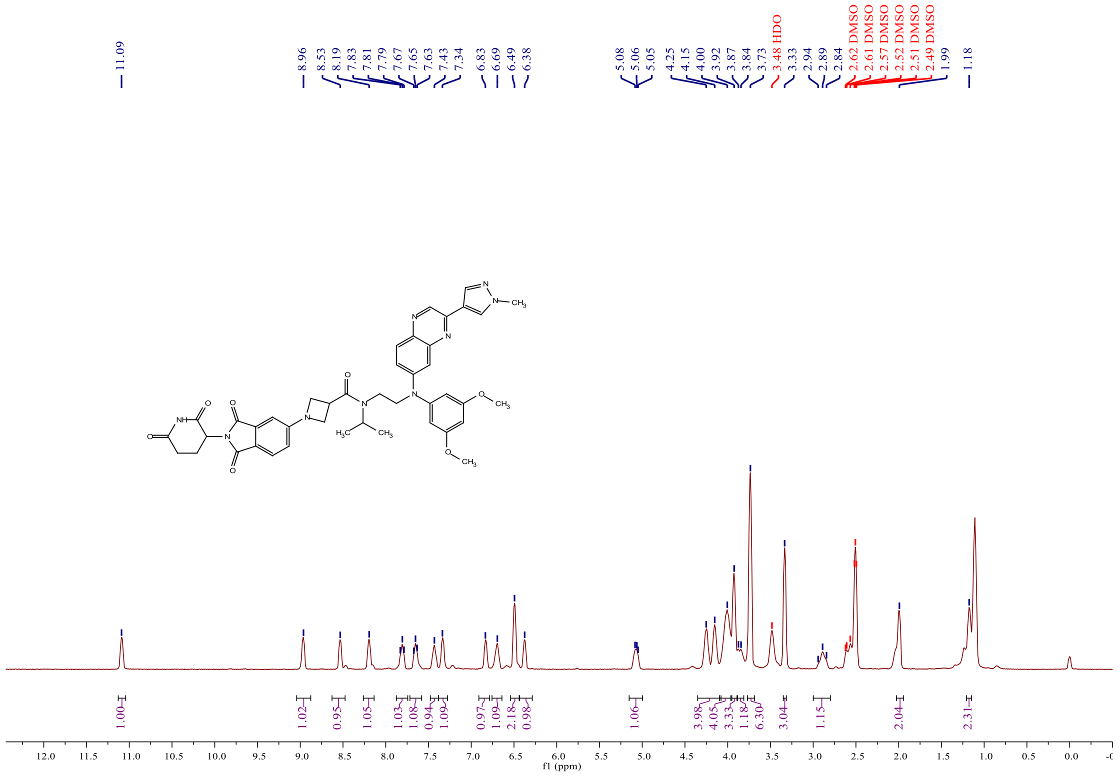

LC-JD-5 ^{13}C NMR

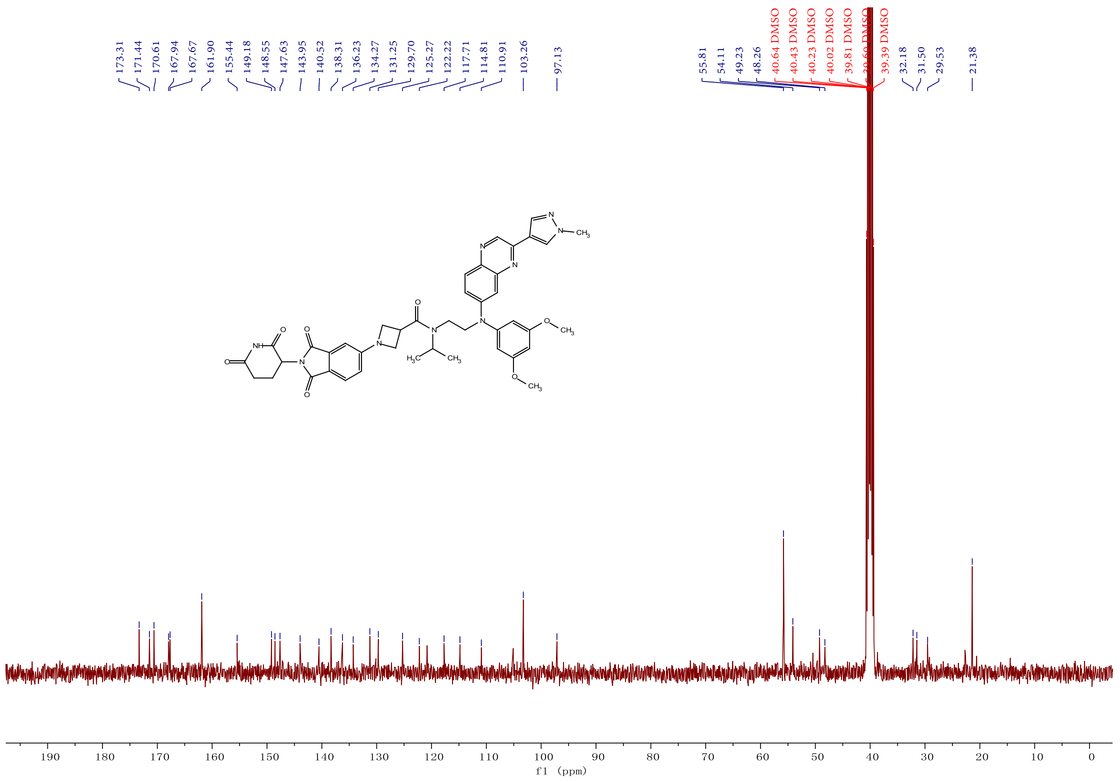

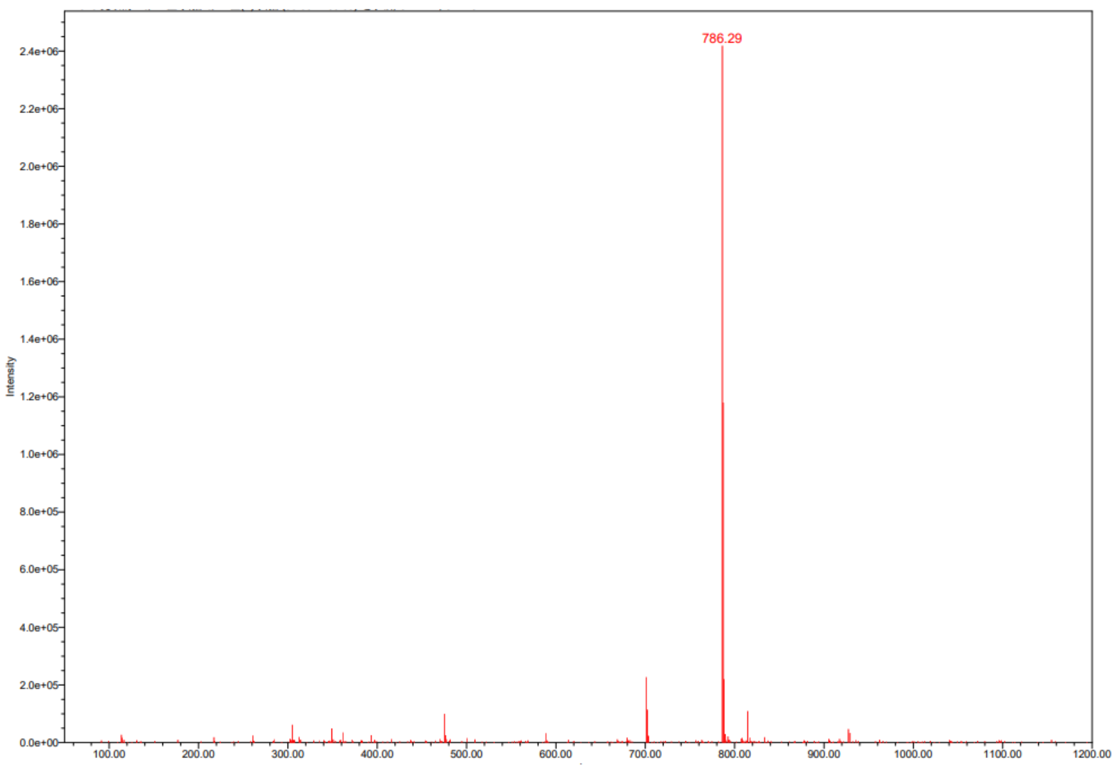

1.6 *N-(2-((3,5-dimethoxyphenyl)(3-(1-methyl-1H-pyrazol-4-yl)quinoxalin-6-yl)amino)ethyl)-1-(2-(2,6-dioxopiperidin-3-yl)-1,3-dioxoisoindolin-5-yl)-N-isopropylpiperidine-4-carboxamide* (LC-JD-6). LC-JD-6 was synthesized in a similar manner as LC-JD-1, yellow powder (52%). ^{1}H NMR (400 MHz, DMSO-d_6) δ 11.15 (s, 1H), 9.03 (s, 1H), 8.60 (s, 1H), 8.26 (s, 1H), 7.88 (dd, *J* = 14.9, 9.1 Hz, 1H), 7.73 (d, *J* = 8.5 Hz, 1H), 7.48 (dd, *J* = 9.2, 2.7 Hz, 1H), 7.40 (d, *J* = 2.3 Hz, 1H), 7.34 (ddd, *J* = 8.7, 5.9, 2.2 Hz, 2H), 7.27 (dd, *J* = 28.6, 2.2 Hz, 1H), 6.55 (d, *J* = 2.2 Hz, 2H), 6.51 (d, *J* = 2.2 Hz, 1H), 6.47 – 6.42 (m, 1H), 5.19 – 5.09 (m, 1H), 4.16 (q, *J* = 5.3 Hz, 4H), 3.98 (s, 3H), 3.91 (s, 1H), 3.80 (d, *J* = 1.3 Hz, 6H), 3.77 (s, 2H), 3.52 – 3.43 (m, 2H), 3.24 (s, 2H), 3.22 (s, 2H), 1.17 (d, *J* = 6.3 Hz, 8H). ^{13}C NMR (101 MHz, DMSO) δ 174.59, 173.32, 170.60, 168.11, 167.46, 162.08, 161.91, 155.35, 155.26, 149.25, 148.44, 147.61, 143.95, 140.45, 138.31, 136.18, 134.58, 131.26, 129.72, 125.53, 122.04, 120.80, 118.10, 110.66, 108.30, 103.76, 103.46, 97.17, 55.82, 49.24, 49.10, 47.90, 47.28, 40.63, 40.42, 40.22, 40.01, 39.80, 39.59, 39.38, 38.11, 31.49, 28.22, 22.70, 21.72, 20.67. LC-MS (ESI$^+$): calcd for $C_{44}H_{47}N_9O_7$ 813.9, 814.42 $[M + H]^+$.

LC-JD-6 ^{1}H NMR

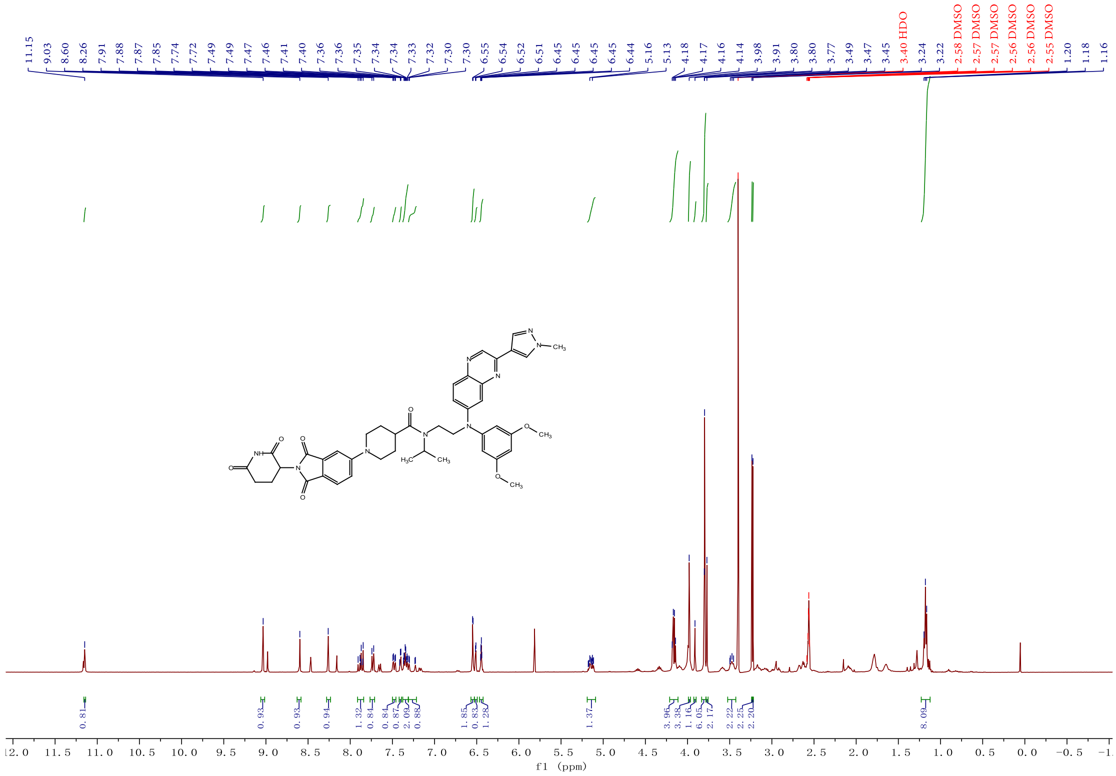

LC-JD-6 ^{13}C NMR

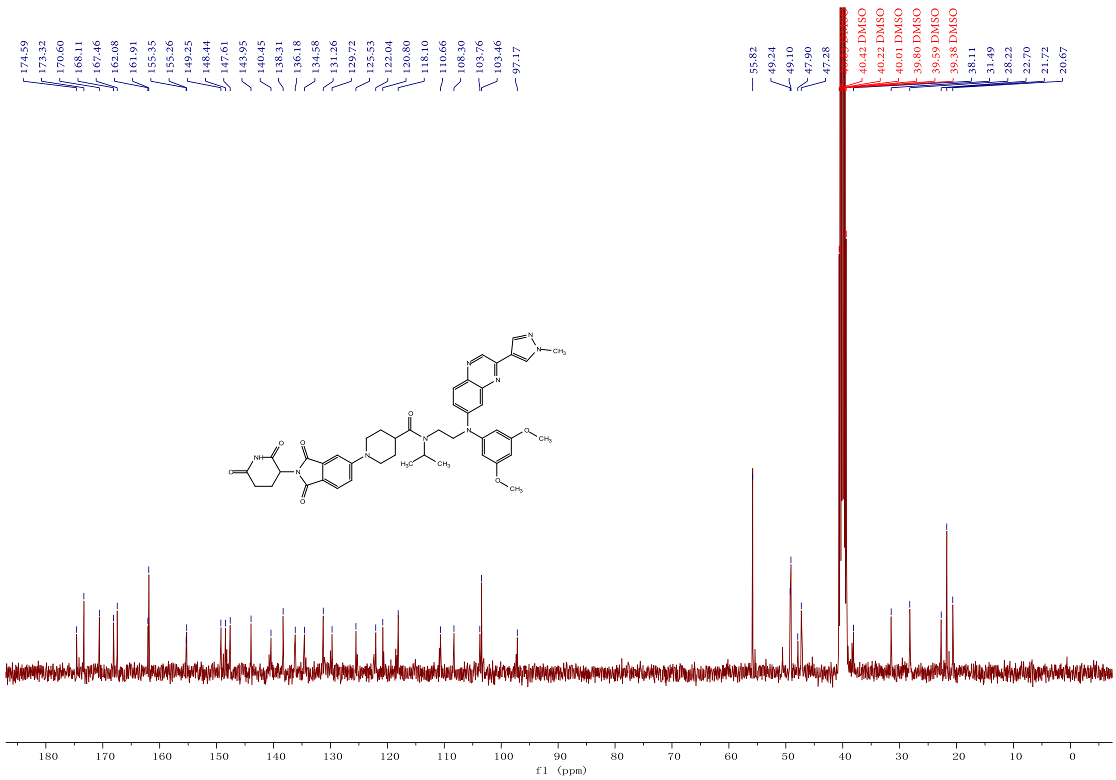

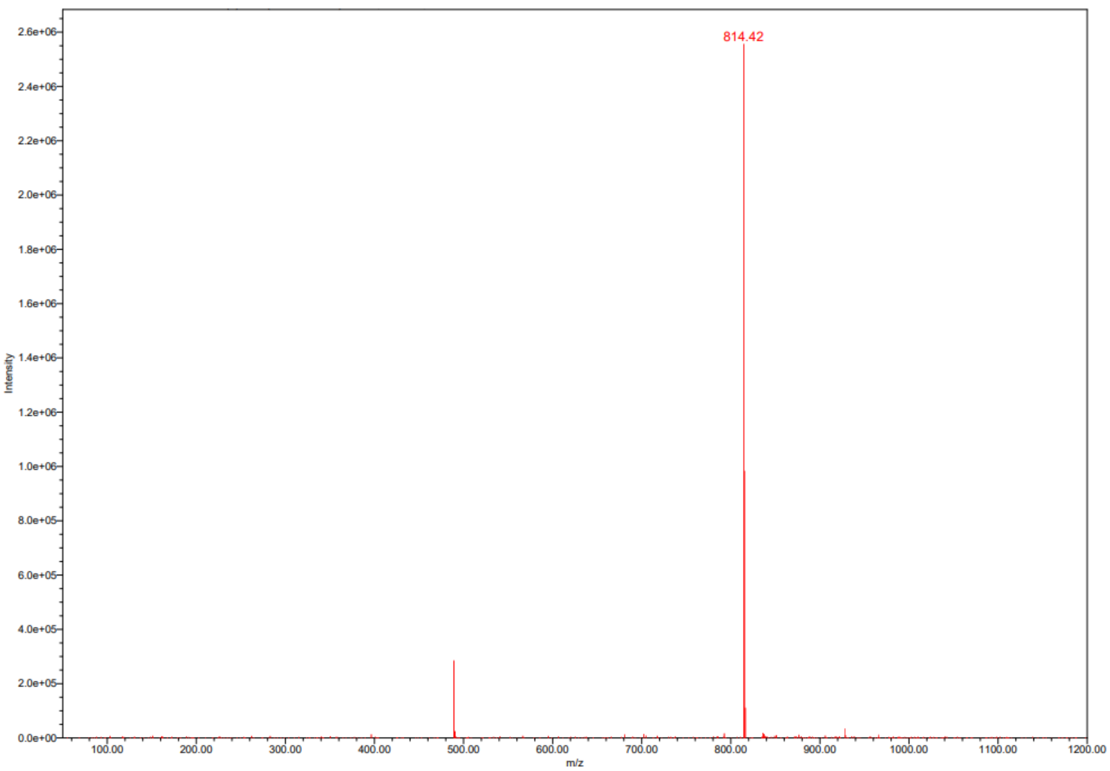

2. Cell culture

RT112, KATO III and NCI-H1581 cell lines were cultured in RPMI 1640 medium supplemented with 10% fetal bovine serum (FBS) and 1% penicillin/streptomycin (P/S). Hep3B was cultured in DMEM medium supplemented with 10% fetal bovine serum (FBS) and 1% penicillin/streptomycin (P/S).

3. Immunoblotting

KATO III cells were seeded into 24-well plates and cultured overnight before treatment with the compounds at various concentrations for the specified time. Cells were collected, washed with phosphate-buffered saline (PBS), and lysed in 2 × loading buffer. The protein samples were subjected to SDS-PAGE gels and transferred to polyvinylidene difluoride (PVDF) membranes, followed by blocking with TBST containing 5% bovine serum albumin (BSA) for 1 h. The membranes were incubated with primary antibodies overnight at 4 °C. The antibodies used in our study were as follows: anti-FGFR1 (1:1000, Cat# 9740S), anti-FGFR2 (1:1000, Cat#11835), anti-FGFR3 (1:1000, Cat# 4574S), anti-FGFR4 (1:1000, Cat# 8562S), anti-p-FGFR (Tyr653/654) (1:1000, Cat# 3471S), were all purchased from Cell Signaling Technology. Anti-Flag-Tag (1:5000, Cat# T0053) was purchased from Affinity Biosciences.

4. Immunofluorescence

To assess the FGFR2 degradation, KATO III cells were treated with 500 nM PA4, fixed with 4% paraformaldehyde for 20 min, permeabilized with 0.3% Triton X-100 in PBS, and blocked with 1% w/v BSA in PBS at room temperature for 30 min. Subsequently, the cells were incubated with anti-FGFR2 antibody (Cat# 4574S, Cell Signaling Technology) overnight at 4 °C, followed by incubation with Alexa Fluor 488-conjugated secondary antibody (Cat# A0423, Beyotime Biotechnology) for 1 h and DAPI (Cat# C1002, Beyotime Biotechnology) for 5 min at room temperature in the dark. Cells were imaged using a Carl Zeiss LSM710 confocal fluorescence microscope.

5. Cell viability assay

The cell viability assay was performed using a luminescent ATP cell viability assay kit (Cat# K2401, APExBIO). Cells were seeded in 96-well plates and treated with the indicated doses of the compounds for 72 h. An equal volume of the luminescent ATP cell viability assay reagent was added and incubated at room temperature for 10 min before measuring the luminescence readings as relative light units (RLU). Cell viability rate (%) was calculated as follows: [(RLU compound − RLU blank)/ (RLU control − RLU blank)] × 100.
